# Visualisation of the Bonebridge by means of CT and CBCT

**DOI:** 10.1186/2047-783X-18-30

**Published:** 2013-09-03

**Authors:** Christian Güldner, Julia Heinrichs, Rainer Weiß, Annette Paula Zimmermann, Benjamin Dassinger, Siegfried Bien, Jochen Alfred Werner, Isabell Diogo

**Affiliations:** 1 University Hospital for ENT, Head and Neck Surgery, Baldingerstraße, 35043, UKGM, Marburg, Germany; 2 University Hospital for Neuroradiology, UKGM, Marburg, Germany; 3 University Hospital for Neuroradiology, UKGM, Gießen, Germany

**Keywords:** Bonebridge, Digital volume tomography (DVT), Cone beam CT (CBCT), Computed tomography (CT), Bone-anchored hearing aid, Magnet resonance imaging (MRI)

## Abstract

**Background:**

With the Bonebridge, a new bone-anchored hearing aid has been available since March 2012. The objective of the study was to analyse the visualisation of the implant itself as well as its impact on the representation of the bony structures of the petrosal bone in CT, MRI and cone beam CT (CBCT).

**Methods:**

The Bonebridge was implanted unilaterally in two completely prepared human heads. The radiological imaging by means of CBCT, 64-slice CT, 1.5-T and 3.0-T MRI was conducted both preoperatively and postoperatively. The images were subsequently evaluated from both the ENT medical and nd radiological perspectives.

**Results:**

As anticipated, no visualisation of the implant or of the petrosal bones could be realised on MRI because of the interactive technology and the magnet artefact. In contrast, an excellent evaluability of the implant itself as well as of the surrounding neurovascular structures (sinus sigmoideus, skull base, middle ear, inner ear, inner auditory canal) was exhibited in both the CT and in the CBCT.

**Conclusion:**

The Bonebridge can be excellently imaged with the radiological imaging technologies of CT and CBCT. In the process, CBCT shows discrete advantages in comparison with CT. No relevant restrictions in image quality in the evaluation of the bony structures of the petrosal bones could be seen.

## Background

The question of the ideal hearing rehabilitation still remains unanswered. Conventional hearing aids, bone-conduction hearing aids, active and passive middle ear implants and cochlea implants as well as combinations of the previously mentioned aids are available. The field of bone-conduction hearing aids presents possibilities of vibration or energy transmission to the cranial calotte by means of pressing on the transducer via arms of glasses or headbands as well as direct anchoring in the bone (bone-anchored hearing aid, BAHA) [1-5]. The drawback of the latter systems is the interruption of the continuity of the skin. This can lead to a higher rate of skin infection as well as to restriction of the wearing comfort [6-14]. Moreover, good osseointegration of the inserted anchor is essential. Another existing system (previously Otomag; subsequent model, Sophono Alpha 1), which leaves the skin intact, has so far not been widely distributed [15].

A consequence of this has been the development of a partially implantable system based on the Vibrant Soundbridge with an audio processor positioned on the skin as well as an implant inserted subcutaneously and into the bone (intact skin technology) in the MedEl^©^ facilities, which was introduced and exhibited officially as the Bonebridge^©^ on the occasion of the ESPO 2012 in Amsterdam. The first implantations were performed in summer 2011, and the data on the initial market introduction studies show excellent audiological results with regard to both pure tone audiometry and language comprehension.

The introduction of new implants always leads to questioning their visualisation by means of cross-sectional diagnostic imaging as well as the impact of the implant on displaying the surrounding structures. For example, in the region of the ear, the bordering neurovascular and brain structures have to be respected as well as ideally further postoperative imaging of the mastoid with organs of the inner ear and structures of the middle ear without influencing the necessary safe diagnostic informational value in relation to these surrounding structures.

Therefore, the aim of this study was to test the visualisation of the implant itself with conventional radiological methods [computed tomography (CT), magnet resonance imaging (MRI), cone beam computed tomography (CBCT)] and to analyse the isolation capability to the surrounding structures of the cranial fossa. The influence of the implant on the visualisation of the surrounding brain structures in the different weightings of the MRI will be reproduced in a separate study for didactic reasons.

## Methods

The Bonebridge^©^ (MedEl, Innsbruck, Austria) was implanted in two deceased body donors post-mortem. To achieve this, both completely prepared heads were freshly defrosted for the first time in order to obtain as accurate a reflexion of the reality of skin flexibility and bone structure as possible. For the estimation of the anatomical structures (sinodural angle, pneumatisation, thickness of the calotte), a CBCT examination (Accu-I-tomo F17, Morita, Kyoto, Japan) was carried out prior to the surgical intervention. Hence, it was possible to plan the desired position of the implant.

**Figure 1:**
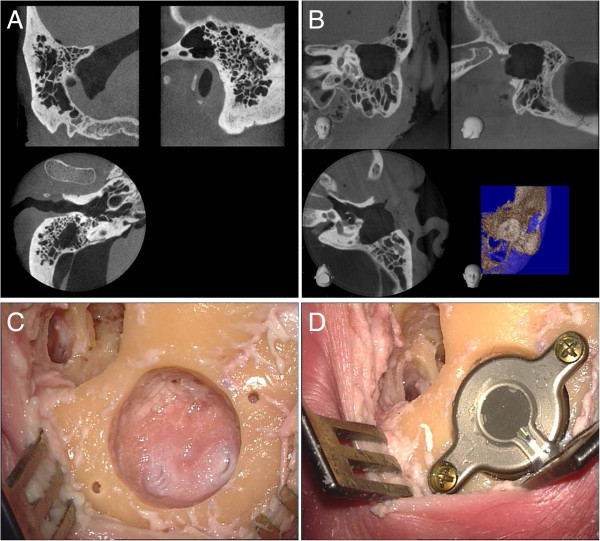
**Preoperative CBCT (A and B) and intraoperative images (C and D).** Preoperative radiological visualisation by CBCT of the site in the case of a well-pneumatised and not pre-operated mastoid **(A)** as well as a site with prior surgery in terms of a transcanalicular partial mastoidectomy **(B)**. Presentation of the site after the drilling of the bed **(C)** and fixation of the implant **(D)**.

The intention was to simulate a classical surgery in the first skull (normal mastoid) (Figure 1A). This was carried out by a retroauricular skin incision parallel to the external ear fold and the preparation of the palva flap. After undermining the periosteum using a raspatorium, it was possible to prepare an adequate pocket as well as to gain an adequate view of the mastoid. The dummy of the ferro-magnetic transducer, the “Bone Conduction-Floating Mass Transducer” (BC-FMT), was positioned in the desired location and marked. The transmission of the radiological planning to the actual surface anatomy proved to be far from a trivial matter. This was followed by the preparation of the mastoid using a sharp drill. Under regular control using the dummy, a correct cylinder with regard to shape and depth was prepared (Figure 1C). In the process, an immediate positional relation to the sinus sigmoideus was shown in the posterior area, however, without injury to its soft tissue casing. The drilling of the holes for the screws was no problem because of the disposable drill with a depth stop included in the delivery. The insertion of the bone conduction implant and the insertion of the magnet into the periosteum pocket also did not present any difficulty. The fixation of the screws using the provided torque wrench did not present any problems (Figure 1D).

In the second skull, the objective was to simulate the potential application in patients following cholesteatoma surgery. To achieve this purpose, a transcanalicular antro-partial mastoidectomy was performed (Figure 1B) for restoration (analogous to the extirpation for example of an extended epitympanalis cholesteatoma following the retroauricular incision). The above-mentioned CBCT imaging was then conducted in order to determine the aspired position of the BC-FMT in this case also. As a result of the previously undertaken surgical steps, the transmission of the position to the surgical site proved to be easier in this case. This was followed by the implantation of the system in the above-described manner. Also in this site, it came in close positional relation to the dura and sinus sigmoideus without injury.

Following the successful implantation, the above-mentioned CBCT device was used for new imaging under the setting parameters established in the course of the daily routine (360° rotation, 84 kV, 8 mA, CTDI = 7.6 mGy) of the implanted ear (target volume of the cylinder: 6 cm height, 6 cm diameter). Furthermore, this was followed by the radiological examination using an in-house CT device (64-slice CT, Siemens, Erlangen, Germany).

Both the skulls were likewise subjected to the following magnetic resonance tomographic (MRI) examinations following the implantation. First, the Siemens Verio whole-body scanner with 3-T field strength with the standard 12-channel head coil (Siemens Healthcare, Erlangen, Germany) and second the Siemens Avanto whole-body scanner with 1.5-T field strength with the standard 12-channel head coil (Siemens Healthcare, Erlangen, Germany) were used. All radiological examinations were performed pre- and postoperatively.

## Results

The position of the implant was determined under exact evaluation of the preoperative CBCT images, whereby the transmission to the actual surgical site was not easy. This was because of the lack of reproducible transfer of the surface structure from imaging to the anatomic situation. One solution and improvement would be to take a navigation system to obtain better matching of imaging and the situation in the operating room. The possibility to insert a BC-FMT 3D template into a volume model of the individual site based on DICOM data allows the preoperative visualisation of the exact location in relation to the anatomical landmarks, whereby the transmission of the planning to the actual intraoperative situation contains hidden sources of error. The surgical steps could be completed quickly and easily under the supervision of the instructors from MedEl^©^. Hence, it was possible to implant Bonebridges in both prepared skulls.

The imaging (CBCT, CT, MRI) was conducted in accordance with the above-defined protocol. No problems were indicated such as dislocation of the implant or of the magnet and coil in MRI in particular. In the clinical evaluation after the conducted MRI examinations (approximately 4 h scanning time for each skull because of the different scanning protocols with the 1.5- and 3.0-T MRI), the implant was shown to be in the same position as before. Functional testing was not performed because of the application of a test implant that was not completely operational. As anticipated, during the analysis of the MRI images, the implant itself could not be evaluated and was not displayed because of complete and excessive artefact radiation.

**Figure 2:**
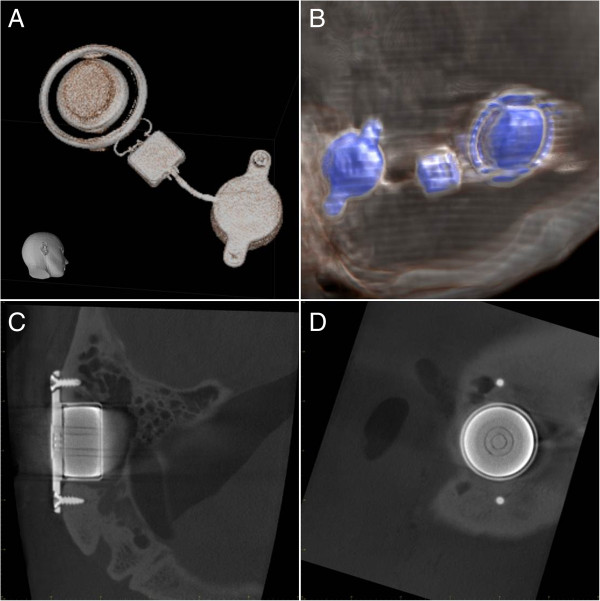
**Visualisation of the implant.** Three-dimensional reconstruction of the implant from the raw data of the CBCT **(A)** and CT **(B)**. Imaging of the implant details including the inner structure and fixing screws in CBCT **(C** and **D)**.

Visualisation of the Bonebridge was easily possible (Figure 2A and B) in both CT and CBCT. Due to the technical prerequisites of CBCT (low target volume, high spatial resolution), it was possible to realise both the structure of the implant itself and the exact visualisation of the fixing screws (Figure 2C and D).

**Figure 3:**
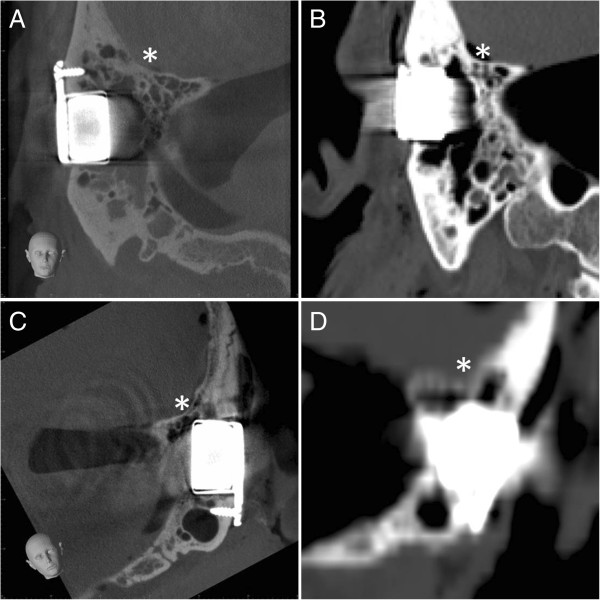
**Positional relation of the implant to the posterior cranial fossa.** Visualisation of the implants in CBCT **(A** and **C)** and in CT **(B** and **D)** for both prepared skulls **(A** and **B**, head 1; **C** and **D**, head 2**)** in relation to the surrounding brain structures (*).

**Figure 4:**
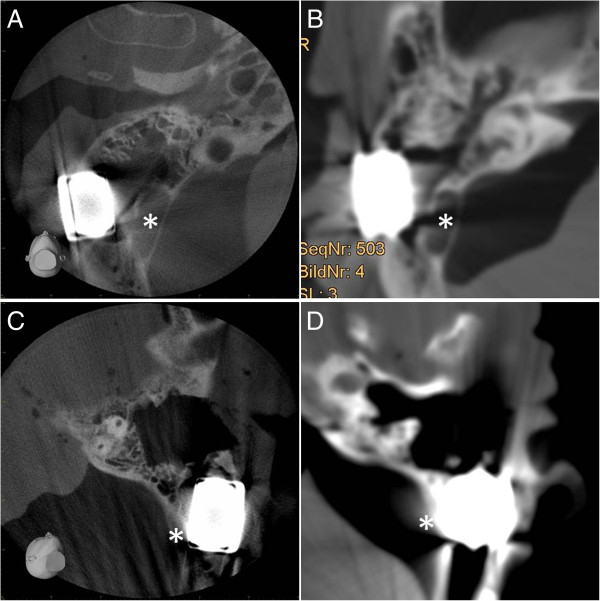
**Positional relation of the implant to the sinus sigmoideus.** Visualisation of the implants in CBCT **(A** and **C)** and in CT **(B** and **D)** for both prepared skulls **(A** and **B**, head 1; **C** and **D**, head 2**)** in relation to the sinus sigmoideus (*).

The analysis of the images in relation to the positional relation of the implant in the bone as well as to the bordering neurovascular structures (posterior cranial fossa, sinus sigmoideus) could basically be conducted in both CT and CBCT (Figures 3 and 4). The image quality, however, showed differences in quality in favour of CBCT. In both the representation of the base of the skull (Figure 3) and the demarcation to the sinus sigmoideus (Figure 4), fewer artefacts and a higher level of image intensity were realised. On observation of the further otoneurologically relevant structures (semicircular canals, inner ear, inner auditory canal), there was no relevant difference in CBCT and CT in the comparison of the pre- and postoperative implantation imaging data.

## Discussion

Ongoing studies with regard to comparison of different bone-conduction hearing aids should demonstrate the respective differential indications. The focus of the current study was the radiological visualisation of the new implant as well as its impact on the imaging of the bony structures in the vicinity of the laterobase.

CT, CBCT and MRI are all currently used in daily cross-sectional imaging diagnostics in the field of ENT medicine. Hence, the question arises for the visualisation of every new implant in the region of the petrosal bone in the three modalities. CT has so far been the gold standard for imaging of the bony structures of the laterobase/petrosal bone. Likewise, CT is the diagnostic choice for queries concerning the neurovascular structures around the implant following the implantation of active implants in the middle or inner ear. CBCT has become increasingly popular in recent years and has been able to show its possibilities in both the visualisation of the laterobase [16-21] and below the frontonasal region [22-26], thus meanwhile presenting an alternative to CT in displaying of bony structures [27].

In this study, it has been possible to generate representative images for displaying the implant itself in CT and CBCT. As anticipated, an artefact-caused overlapping of the implant is shown on MRI, meaning that no visualisation of the implant or the directly bordering anatomical structures was possible. Excellent visualisation of the 3D structure of the implant was achieved in both CT and CBCT. A good presentation of the surrounding, surgically significant anatomical structures (inner ear, semicircular canals, inner auditory canal, rear skull base, sinus sigmoideus) were also shown, whereby in the case of CBCT, the image quality in relation to the bony structures tended to be better. All in all, a visualisation around the implant is possible in both modalities, meaning that in the case of a new surgical intervention in the region of the petrosal bone, statements relating to the anatomical circumstances can be made and therefore preoperative planning can be achieved. A big advantage of CT in combination with contrast media is the possibility of visualisation of soft tissues. Therefore, CT could be an alternative to MRI, where the inner auditory canal is overlapped by artefacts, in visualisation of the bony and soft tissue structures of the inner auditory canal in case of radiological control after implantation in case of hearing loss after diseases of this structure (e.g. acoustic neuromas).

## Conclusion

This is the first study describing the radiological aspects of the brand-new bone-conducting implant “Bonebridge”. Visualisation of the implant itself and the surrounding anatomical structures is possible with CT and CBCT as well. Regarding imaging quality, CBCT shows advantages in comparison to conventional CT.

## Abbreviations

BAHA: Bone-anchored hearing aid; BC-FMT: Bone conduction-floating mass transducer; CBCT: Cone beam computed tomography; CT: Computed tomography; CTDI: Computed tomography dosage index; DVT: Digital volume tomography; ESPO: European Society of Paediatric Otolaryngology; ENT: Ear-nose-throat; MRI: Magnet resonance imaging.

## Competing interests

The authors declare that they have no competing interests.

## Authors’ contributions

CG planned the study, carried out the operations, accompanied the imaging and wrote the manuscript. JH carried out the imaging procedures of CT. RW participated in the study design and at operating procedures. AZ participated at CBCT imaging, BD carried out the MR imaging, SB participated at CT imaging, JAW participated in the study design and coordination. ID carried out the CBCT imaging procedures and helped to draft the manuscript. All authors read and approved the final manuscript.
